# Supplementary material for: Xenon iron oxides predicted as potential Xe hosts in Earth’s lower mantle
Source: Nat Commun. 2020 Oct 16;11:5227. doi: 10.1038/s41467-020-19107-y (PMC7568531; doi:10.1038/s41467-020-19107-y)
Supplement: Supplementary file 1 — Supplementary Information [file 41467_2020_19107_MOESM1_ESM.pdf]

Supplementary information

for

Xenon Iron Oxides Predicted as Potential Xe Hosts in Earth's Lower Mantle

Feng Peng,[1,2,$] Xianqi Song,[3,4,$] Chang Liu,[5] Quan Li,[3,5*] Maosheng Miao,[2] Changfeng Chen,[6*] and Yanming Ma[3,4,7*]

[1]*College of Physics and Electronic Information & Henan Key Laboratory of Electromagnetic Transformation and Detection, Luoyang Normal University, Luoyang 471022, China*

[2]*Department of Chemistry and Biochemistry, California State University Northridge, Northridge, CA, 91330-8262, USA*

[3]*State Key Laboratory of Superhard Materials, College of Physics, Jilin University, Changchun 130012, China*

[4]*Innovation Center for Computational Physics Methods and Software, College of Physics, Jilin University, Changchun 130012, China.*

[5]*Key Laboratory of Automobile Materials of MOE and Department of Materials Science, College of Materials Science and Engineering, Jilin University, Changchun 130012, China*

[6]*Department of Physics and Astronomy, University of Nevada, Las Vegas, Nevada 89154, USA*

[7]*International Center of Future Science, Jilin University, Changchun 130012, China*

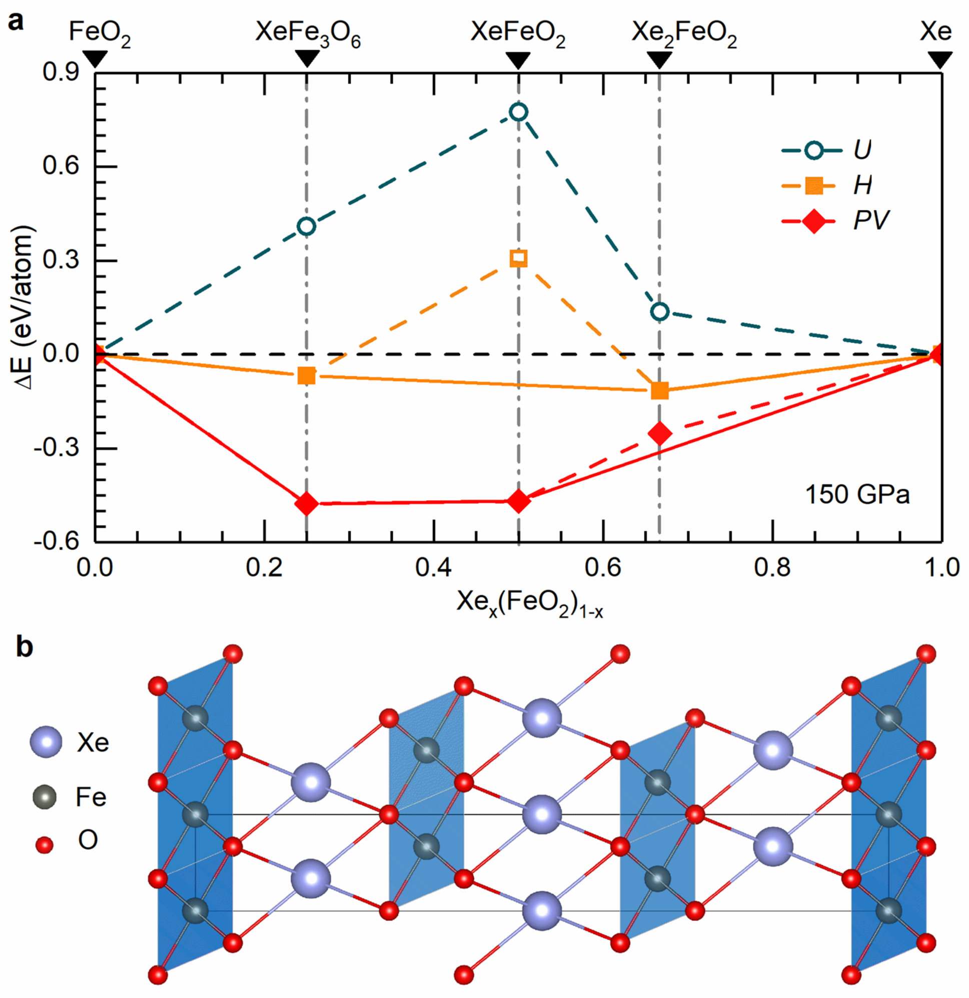

Supplementary Figure 1. Calculated energetic terms of FeO_2-Xe compounds and the crystal structure of $XeFeO_2$ at 150 GPa. a The enthalpies ΔH, the internal energies ΔU, and the pressure-volume term $\Delta(PV)$ for the Xe-Fe oxides. b The polyhedral views of theoretically predicted $XeFeO_2$ structure. We discuss the energetic terms to examine the mechanism of thermodynamic stability for these FeO_2-Xe compounds. The pressure-volume terms, associated with packing efficiency, make similar contributions to lowering the enthalpy for $XeFe_3O_6$ and $XeFeO_2$, while the unfavorable internal energy, associated with bonding enhancement, leads to a positive formation enthalpy for $XeFeO_2$. Although the value of pressure-volume term of Xe_2FeO_2 is much lower than these of $XeFe_3O_6$ and $XeFeO_2$, it still offsets the relatively weak negative effect of internal energy to enthalpy, thus yielding its thermodynamic stability with formation enthalpy lying on the convex hull.

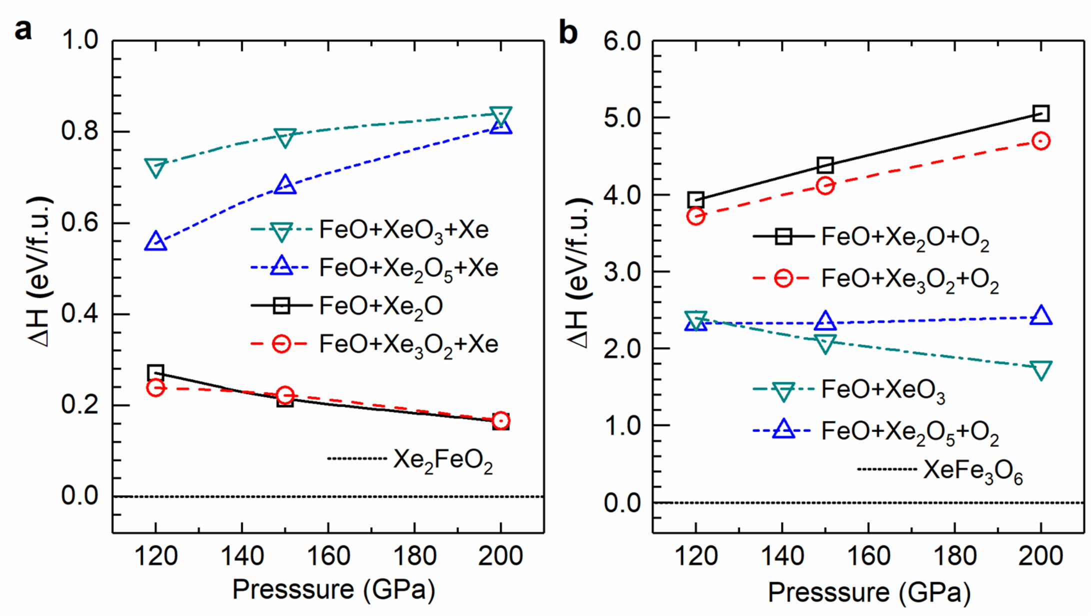

Supplementary Figure 2. The relative stability of Xe_2FeO_2 and $XeFe_3O_6$ relative to FeO and Xe oxides, respectively. The results in Supplementary Figure 1a shows that Xe_2FeO_2 is at least 0.165 eV/f.u. more stable in energy relative the products of FeO + Xe_2O, FeO + Xe_3O_2 + Xe, FeO + Xe_2O_5 + Xe, and FeO + Xe_3O_2 + Xe. For $XeFe_3O_6$, the results in Supplementary Figure 2b shows that it is at least 1.749 eV/f.u. energetically much superior and against decomposition into a mixture of FeO + Xe_2O+ O_2, FeO + Xe_3O_2 + O_2, FeO + Xe_2O_5 + O_2, or FeO + XeO_3.

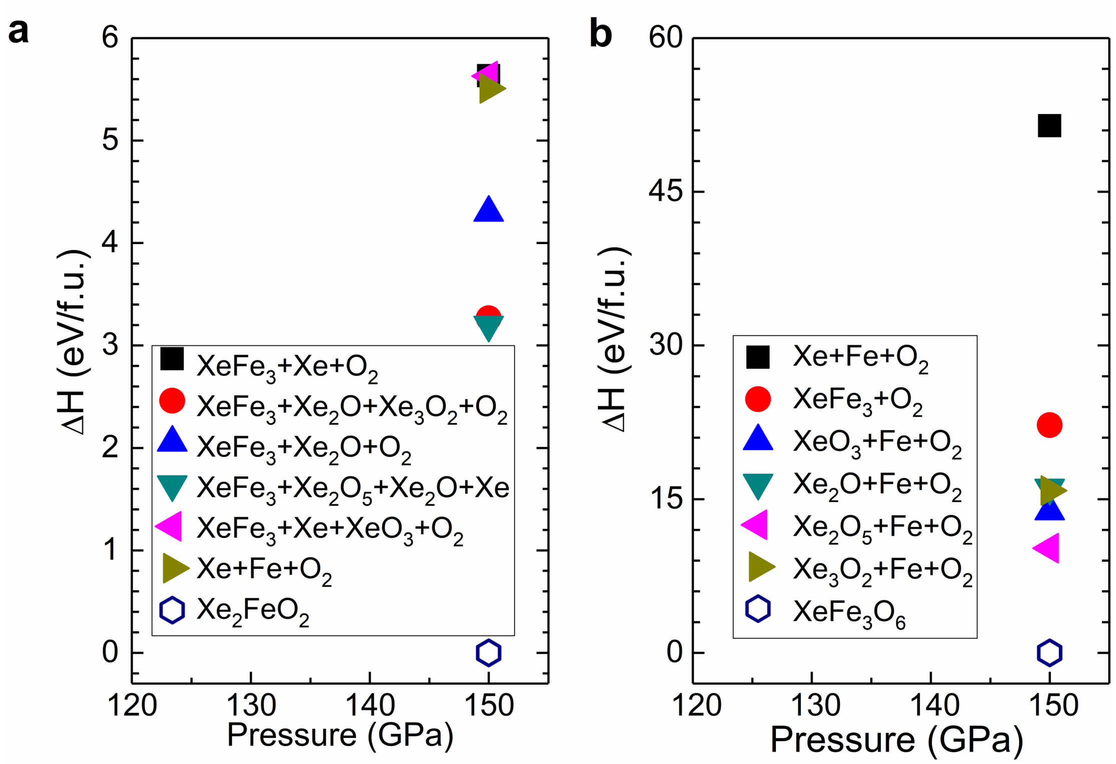

Supplementary Figure 3. The relative stability of Xe_2FeO_2 and $XeFe_3O_6$ relative to Xe_xFe_y/Fe and O/Xe oxides, respectively. Our results show that Xe_2FeO_2 [Supplementary Figure 3a] is at least 3.265 eV/f.u. in energy more stable than the products of $XeFe_3$ + Xe + O_2, $XeFe_3$ + Xe_2O + O_2, $XeFe_3$ + Xe_3O_2 + Xe_2O +O_2, $XeFe_3$ + Xe_2O_5 + Xe_2O +Xe, $XeFe_3$ + XeO_3 +Xe + O_2, or Fe + Xe + O_2. Supplementary Figure 3b reveals that $XeFe_3O_6$ remains stable (even reach ~10.0 eV/f.u.) relative the products of Fe + Xe + O_2, $XeFe_3$ + O_2, Fe + XeO_3 +O_2, Fe + Xe_2O_5 + O_2, Fe + Xe_3O_2 + O_2, or Fe + Xe_2O + O_2.

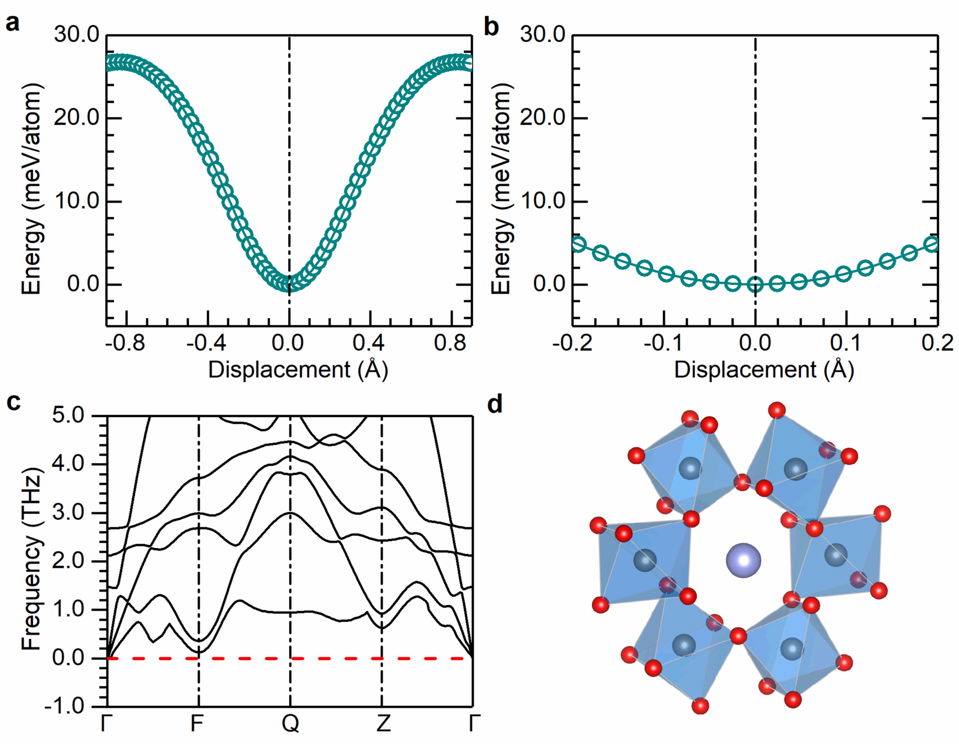

Supplementary Figure 4. The vibrational analyses for $XeFe_3O_6$. a The internal energy difference at various Xe positions along the channels (the vibration eigenvector of the vibrational mode of the lowest-frequency) of the Fe-O framework at 150 GPa and zero temperature. The original structure is located at an energy minimum on the energy surface, signifying its dynamical stability. b The internal energy difference of $XeFe_3O_6$ at various Xe positions along the channels near the equilibrium position. c The theoretical phonon spectrum at the low-frequency region. d The snapshot for the Fe-O framework with Xe atom in the channel. The vibrational modes close to zero for $XeFe_3O_6$ stem from the relatively large internal volume for Xe atom that leads to the concomitant longer bond lengths and weak interaction between Xe atoms and O atoms. The maximum distance between Xe and O atom is 2.52 Å in $XeFe_3O_6$ at 150 GPa, which is much longer than that of Xe oxides (e.g. ~2.37 Å in Xe_2O_5) and Xe_2FeO_2 with CN=3 of Xe (2.42 Å) at the same pressure. As a result, Xe-O bonds of Xe_2FeO_2 are much stronger than that of $XeFe_3O_6$. To explore the possible dynamical instabilities, we have move one Xe atom along the channels of the Fe-O framework (the vibration eigenvector of the mode for the lowest-frequency branch at F) while fixing the other atoms. The calculated static energy shows that the original structure is located at an energy minimum on the energy surface, signifying its dynamical stability. The curve is smooth near the equilibrium position (e.g., < 0.2 Å), making it easier for the Xe atoms to move about along the eigenvector of the low-frequency phonon mode, in agreement with the theoretical phonon spectrum. Meanwhile, the curve has a rather high energy cost at large displacements, and the phase transition is impeded by kinetic barrier of ~26.8 meV/atom at 150 GPa and zero temperature, indicating that it is difficult for the Xe atoms to escape from the lattice. With the consideration of temperature effect, our AIMD calculations also reveal that Xe atoms can only vibrate near the balanced positions below melting points.

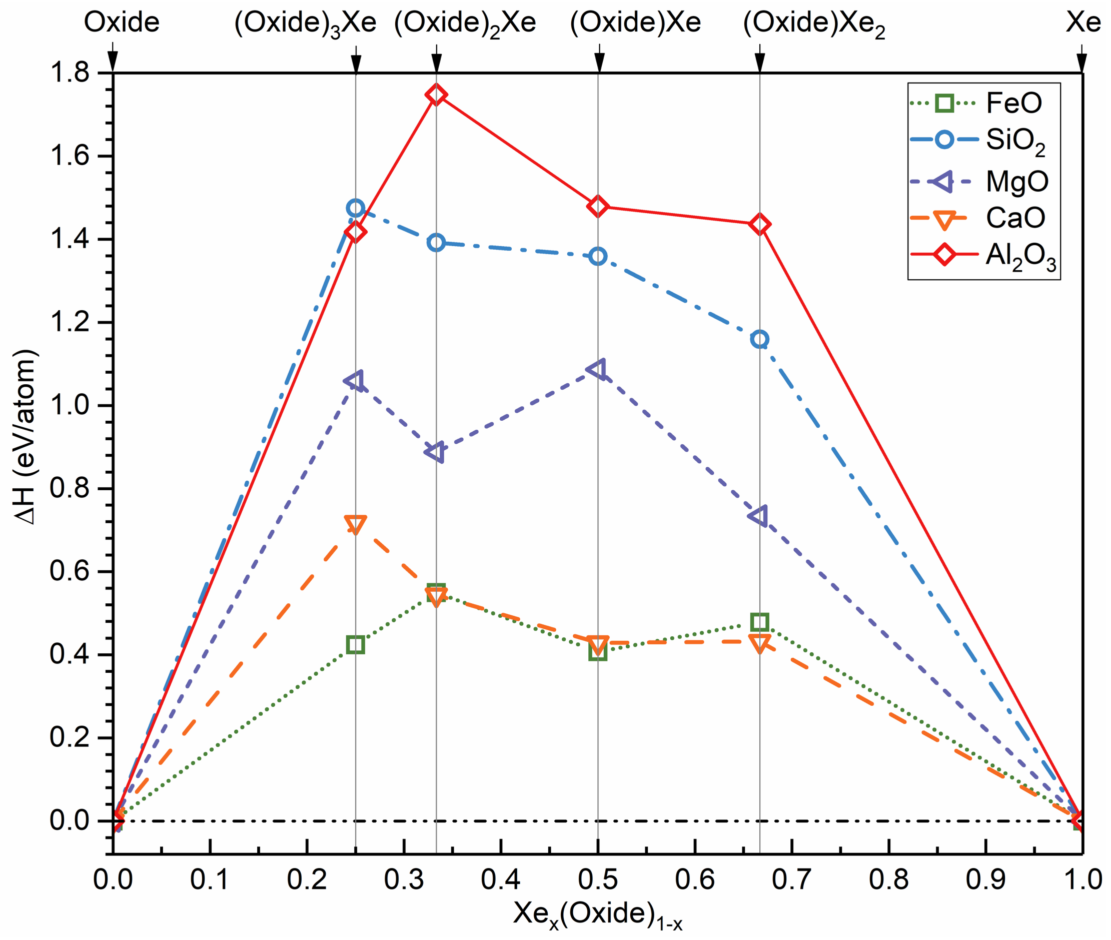

Supplementary Figure 5. Enthalpies of formation of various Oxide-Xe (Oxide = FeO, SiO_2, MgO, CaO or Al_2O_3) compounds at 200 GPa. All the Oxide-Xe compounds which have positive formation enthalpies are not stable at 200 GPa. (Correlation effects among the Fe 3d electrons were treated in the GGA + *U* approach, adopting the recently proposed on-site Coulomb interaction U = 5.0 eV and a Hund's coupling J = 0.8 eV and the spin-polarized and magnetic states are considered to calculate the formation enthalpies.)

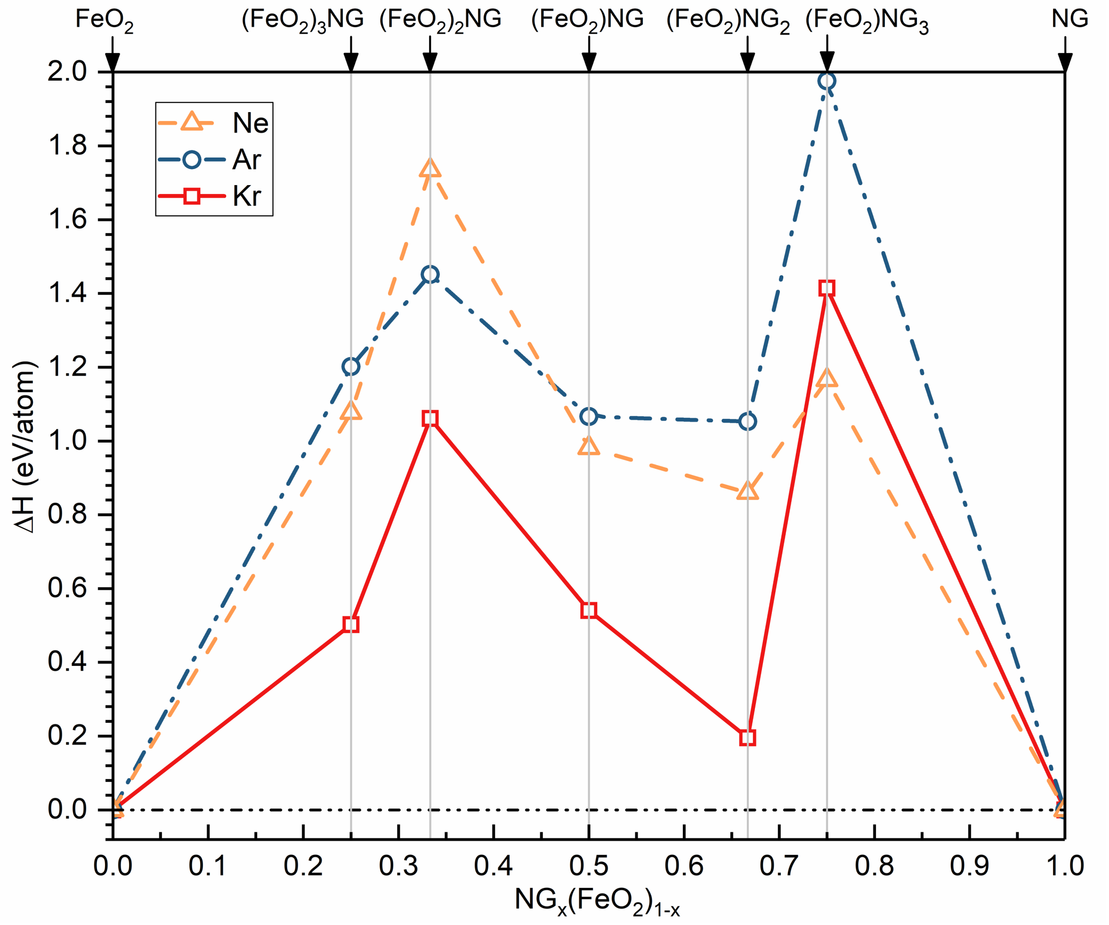

Supplementary Figure 6. Enthalpies of formation of various FeO_2-NG (NG=Ne, Ar, Kr) compounds at 200 GPa. All the FeO_2-NG compounds which have positive formation enthalpies are not stable at even 200 GPa.

Supplementary Table 1. Calculated structural parameters of $XeFe_3O_6$ and Xe_2FeO_2.

	Space group	Lattice Parameters (Å, °)	Atomic coordinates (fractional)			
			**Atoms**	***x***	***y***	***z***
$XeFe_3O_6$	*P*-1	*a* = 5.043	Fe1(2i)	0.7501	0.0025	0.5003
(150 GPa)		*b* = 5.702	Fe2(2i)	0.2503	0.4999	0.0014
		c = 5.780	Fe3(1d)	0.5000	0.0000	0.0000
		α = 119.475	Fe4(1a)	0.0000	0.0000	0.0000
		β = 95.276	O1(2i)	0.7114	0.7456	0.9008
		γ = 96.930	O2(2i)	0.7866	0.2544	0.0979
			O3(2i)	0.9452	0.3337	0.7559
			O4(2i)	0.4869	0.0716	0.3269
			O5(2i)	0.5565	0.6692	0.2431
			O6(2i)	0.0113	0.9333	0.6761
			Xe(2i)	0.2500	0.4979	0.5014
Xe_2FeO_2	$P2_1/c$	*a* = 11.100	Fe1(4e)	0.0003	0.5086	0.7496
(150 GPa)		*b* = 5.584	Fe2(4e)	0.0001	0.7501	0.4994
		c = 9.544	O1(4e)	0.8919	0.2600	1.0763
		α = 90.000	O2(4e)	0.1085	0.2590	0.5776
		β = 136.089	O3(4e)	0.8928	0.0025	1.3271
		γ = 90.000	O4(4e)	0.1070	0.4958	0.3271
			Xe1(4e)	0.6567	0.5036	0.7971
			Xe2(4e)	0.3420	0.7476	0.0480
			Xe3(4e)	0.3438	0.2477	0.0468
			Xe4(4e)	0.6571	0.0033	0.7974
$XeFeO_2$	*R*-3*m*	*a* = *b* = 2.676	Fe (3a)	0.0000	0.0000	0.0000
(150 GPa)		*c* = 16.629	O (6c)	0.0000	0.0000	0.2794
		α = *β* = 90.000	Xe (3b)	0.0000	0.0000	0.5000
		γ = 12.000				
